# Advances in macrophage-myofibroblast transformation in fibrotic diseases

**DOI:** 10.3389/fimmu.2024.1461919

**Published:** 2024-10-09

**Authors:** Jia-Qi Ban, Li-Hong Ao, Xiu He, Hua Zhao, Jun Li

**Affiliations:** School of Public Health, the Key Laboratory of Environmental Pollution Monitoring and Disease Control, Ministry of Education, Guizhou Medical University, Guiyang, China

**Keywords:** macrophage, macrophage-myofibroblast transition, fibrosis, TGF-β, myofibroblast

## Abstract

Macrophage-myofibroblast transformation (MMT) has emerged as a discovery in the field of fibrotic disease research. MMT is the process by which macrophages differentiate into myofibroblasts, leading to organ fibrosis following organ damage and playing an important role in fibrosis formation and progression. Recently, many new advances have been made in studying the mechanisms of MMT occurrence in fibrotic diseases. This article reviews some critical recent findings on MMT, including the origin of MMT in myofibroblasts, the specific mechanisms by which MMT develops, and the mechanisms and effects of MMT in the kidneys, lungs, heart, retina, and other fibrosis. By summarizing the latest research related to MMT, this paper provides a theoretical basis for elucidating the mechanisms of fibrosis in various organs and developing effective therapeutic targets for fibrotic diseases.

## Highlights

- MMT plays a crucial role in the development of fibrotic diseases.
- Mechanism of macrophage to myofibroblast transformation.
- Modulation of MMT is expected to prevent and treat fibrosis.

## 1 Introduction

Fibrosis is the excess buildup of extracellular matrix components, resulting in tissue structure damage and loss of organ function. This condition can impact any organ, with fibrosis contributing to 45% of global deaths (1). The high morbidity and mortality rates worldwide are attributed to the limited availability of effective fibrosis treatment options (2). Fibrosis has traditionally been viewed as irreversible; however, both preclinical models and clinical trials have revealed that fibrosis is a very dynamic process. Multiple studies indicate that various mediators stimulate fibre formation, indicating that focusing on these mediators could be a successful therapeutic approach for combating fibrosis. Therefore, exploring fibrosis pathogenesis and identifying effective therapeutic targets remain the primary focus of current research. Macrophages and fibroblasts are important for the development of fibrosis. Macrophages release many proinflammatory cytokines and chemokines that trigger an inflammatory response and damage tissue. In addition, they crosstalk with fibroblasts and promote the differentiation of fibroblasts into myofibroblasts, which secrete large amounts of collagen, leading to organ fibrosis (3–5). Thus, investigating the connection between macrophages and fibroblasts could aid in gaining deeper insight into the precise mechanisms of fibrogenesis.

A novel mechanism, MMT, has recently been identified as a critical component of organ fibrosis in response to chronic inflammation. MMT is the process by which bone marrow-derived macrophages (BMDMs) differentiate into myofibroblasts and lead to organ fibrosis after organ injury (6). Myofibroblast-like cells (MMT cells) can produce collagen and can be distinguished by the co-expression of the macrophage marker CD68 and the myofibroblast marker a-SMA (7). In addition to macrophages, a diverse array of cellular constituents, including fibroblasts, epithelial cells, endothelial cells, mesothelial cells, and pericytes, have been found to play important roles in the development and progression of organ fibrosis (8). The enigmatic process of MMT has garnered significant attention in recent years as a focal point in unravelling the pathogenic underpinnings of organ fibrosis (3, 9, 10). However, what are the specific mechanisms by which MMT occurs? Its role in the development of fibrotic diseases has not been fully characterized. Therefore, this paper explores how MMT occurs, the conditions under which it occurs, its involvement in organ fibrosis formation, and its specificity in each organ. Recent evidence and advancements in the mechanisms of MMT and its role in fibrotic diseases are outlined. Understanding this phenomenon and its associated signalling pathways will help to identify therapeutic targets for fibrotic diseases.

## 2 Origin of myofibroblasts

Myofibroblasts are a subset of activated fibroblasts and are the main cells responsible for excess extracellular matrix (ECM) in organ fibrosis (11). Despite myofibroblasts being discovered more than 50 years ago, more information has been gathered about their involvement in wound healing and fibrosis. The origin of myofibroblasts, however, remains contentious, with numerous progenitor cells being suggested (12). Initially, thought to be in a terminal stage of differentiation, recent findings indicate that myofibroblasts are transient and reversible (13). Additionally, myofibroblasts possess a wider range of adaptability and can change into other specialized mesenchymal lineages once the injury heals. For instance, myofibroblasts in rat skin wounds cease their contractile activity and are transformed into new fat-filled adipocytes in reaction to bone morphogenetic protein (BMP) ligands released by hair follicles (14). The transitions in the state of myofibroblasts are essential for tissue recovery after injury, yet their abnormal and persistent conversion significantly contributes to fibrosis and promotes cancer progression (15).

Myofibroblasts are a diverse group of active fibroblasts that can undergo various transformations, such as epithelial–mesenchymal transition (EMT) (15, 16) from epithelial cells, macrophage-myofibroblast transition (MMT) from bone marrow-derived macrophages, and endothelial-mesenchymal transition (EndoMT) (17) from endothelial cells, as well as from resident fibroblasts and bone marrow-derived monocytes. These transformations play a vital role in the occurrence of fibrosis (18). During EMT, epithelial cells release epithelial markers (E-calmodulin and occludin) and acquire mesenchymal markers (FSP-1, α-SMA, N-calmodulin, and fibronectin). Research found that TGF-β superfamily signaling can induce fibroblast proliferation and lineage transitions by other cells toward a fibroblast state, including via EMT in the lung (15). In the heart, epicardial cells generate fibroblasts through epithelial-to-mesenchymal transition (EMT) (19). Similarly, during EndoMT, endothelial cells lose endothelial markers (CD31 and VE-calmodulin) and acquire mesenchymal markers. However, during MMT, myofibroblasts exhibit both M2 phenotypic macrophage labelling (CD206) and mesenchymal labelling. Signalling pathways such as the TGF-β, BMP, and Wnt pathways are essential for activating mesenchymal transition. Many studies have investigated the mechanisms of collagen accumulation and the properties of myofibroblasts under fibrotic conditions (20, 21). Bone marrow MSCs exert anti-inflammatory and antifibrotic effects in several disease models (22, 23). Conversely, bone marrow-derived fibroblasts and macrophages have been suggested to be fibroblast precursor cells for renal fibrosis (24–28). An in-depth examination of genetically modified mice with renal fibrosis revealed that the myofibroblast population splits into two categories: 50% from resident fibroblast proliferation and nonproliferating myofibroblasts derived from transdifferentiated MMT (35%) from bone marrow MSCs/stromal cells, EndoMT (10%), and renal tubular EMT (5%) (29). However, nearly 50% of myofibroblasts in tissue fibrosis originate from bone marrow-derived macrophages (30). Although the proportion found varies, it can be found that MMT accounts for a large proportion of myofibroblasts, implying that MMT is an important source of myofibroblasts, which substantially contributes to the development and progression of tissue fibrosis (31).

## 3 MMT

MMT is a new term created in 2014 that describes how BMDMs differentiate into myofibroblasts and promote organ fibrosis during organ injury (32). In recent years, multiple studies have verified the existence of MMT in different systems, indicating that MMT could be present in all fibrotic diseases and offers potential as a viable therapeutic target for halting the progression of fibrotic diseases (12). Monocytes are the primary cells in which MMT occurs. They are the natural precursors of macrophages, which are highly plastic circulating cells of the bone marrow (33). Monocytes can transform into other cells through processes such as monocyte-to-endothelial cell transformation (20), monocyte-to-dendritic cell transformation (21), monocyte-to-macrophage transformation, and further transformation of transformed macrophages to myofibroblasts (MMT).

Macrophages, especially bone marrow-derived macrophages, are key cells in the MMT process (32). They play a crucial role in innate immune defense and maintaining organ homeostasis in a tissue-specific manner (34). Activated macrophages are essential for the production of TGF-β1, a cytokine that plays a dual role in healing and fibrosis depending on its activation timing and location (35). Macrophage recruitment is critical for normal tissue repair, but prolonged macrophage recruitment postacute restoration can lead to excessive tissue repair and fibrosis (36). Cells of the macrophage lineage are highly heterogeneous because of the fact that their functional responses are strongly influenced by the local microenvironment (37, 38). They serve as sentinels in pathogen elimination and tissue regeneration and can also contribute to tissue damage (39). Through phenotypic changes in response to microenvironmental signals, macrophages can polarize into proinflammatory or anti-inflammatory phenotypes. *In vitro* studies have found that under the influence of LPS and IFN-γ, macrophages adopt the M1 subtype characterized by the surface markers CD86 and iNOS, releasing proinflammatory cytokines such as TNF-α, IL-1β, and IL-6. Conversely, activation by IL-4 and IL-13 leads to the M2 phenotype expressing the surface markers CD206, CD163, and Arg-1. M2 macrophages release high levels of IL-10 and TGF-β, which promote tissue healing, fibrosis, and angiogenesis, along with the proliferation of myofibroblasts (40). *In vitro* research has indicated that M1 cells are responsible for the phenomenon of MMT in biology, primarily through the STAT1/STAT3 axis (7, 41). MMT is believed to occur in macrophages when they receive initial inflammatory signals (M1 polarization) followed by anti-inflammatory signals. This finding suggested that M1-type macrophages may play a role in MMT (42). Previous studies have shown that under hypoxic conditions, MMT macrophages are of the M1 type, possibly linked to early kidney injury stages (43). An analysis of MMT responses using cultured bone marrow macrophages revealed a more pronounced reaction in macrophages expressing M2 markers than in those expressing M1 markers, indicating that bone marrow-derived M2 macrophages have a greater MMT capacity than M1 macrophages (44). There is still some debate about the primary macrophage subtype involved in MMT (45, 46). However, most current research indicates that M2 macrophages are the subtype undergoing MMT, with nearly 80% of MMT cells derived from the M2 subpopulation and regulated by TGF-β/Smad3 signalling (7). However, further studies are needed to confirm these findings.

**Figure 1:**
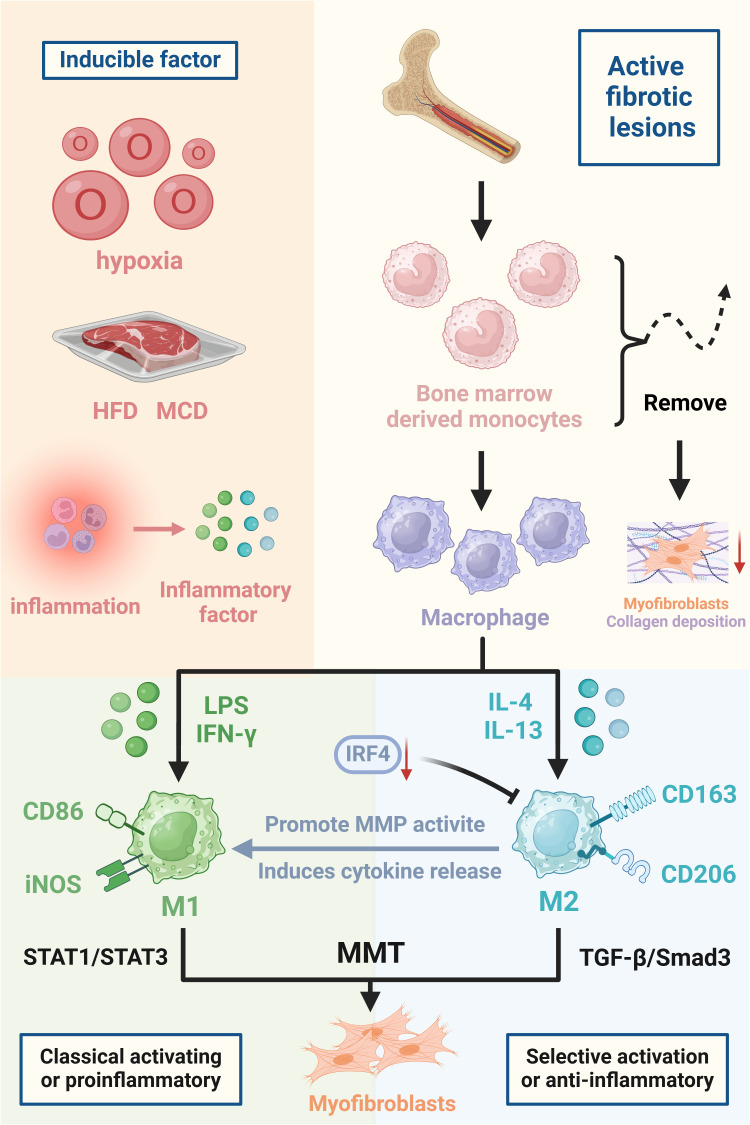
Triggers and major cells involved in the development of MMT. The figure focuses on the triggers of MMT, which are hypoxia, high-fat diet (HFD), methionine choline deficiency diet (MCD), and inflammatory factors released in an inflammatory milieu all predispose to the development of MMT. The differentiation of BMDMs into myofibroblasts and promotion of organ fibrosis during organ injury. Macrophages polarised to M1 and M2 types, inducers of MMT, cell surface markers and mechanistic pathways are also indicated in the figure. Created with BioRender.com.

A variety of factors can influence the development of MMT. After recruitment to damaged tissue, monocytes can differentiate into various macrophage phenotypes. Macrophages can assume various phenotypes under normal physiological conditions, especially in tissue injury scenarios where the local microenvironment is affected by pathogens, cellular damage, innate and adaptive immune responses, hypoxia, and tissue repair processes (3). Persistent macrophage accumulation in damaged organs eventually becomes pathological, leading to irreversible fibrosis, tissue destruction and progressive chronic disease (47). MMT cells are present in fibrotic lesions that are actively progressing but are mostly not found in lesions that are in the acute inflammatory or sclerotic stage (7). TGF-β1, which is secreted mainly by activated macrophages, is one of the key factors contributing to the development of fibrosis, and it is thought to be one of the main factors that induces MMT development (6). TGF-β1 is able to induce collagen-producing MMT cells in bone marrow-derived macrophages, although macrophages may also be involved in the internalisation and degradation of collagen in disease states (31). The study found that IL-22 promotes fibrosis by enhancing the effect of TGF-β and may be involved in the proliferation of myofibroblasts and the occurrence of MMT (48). The microenvironment is critical for initiating MMT in chronic inflammatory diseases, including cancer (49). MMT is found in both high-fat diet (HFD)- and methionine choline-deficient diet (MCD)-producing non-alcoholic fatty liver disease (NAFLD) animals, as well as in ethanol-induced alcoholic fatty liver disease (AFLD) models (50). Removing bone marrow-derived lineage cells hindered the development of MMT cells and notably decreased the formation of myofibroblasts and collagen accumulation in experimental renal fibrosis (7). In conclusion, the triggering factors of MMT are very complex, including cellular factors, the inflammatory environment and dietary metabolism, and the specific underlying mechanisms still need to be further explored (**Figure 1**).

## 4 MMT and fibrotic diseases

The MMT is a complex process that is crucial for the development of fibrotic diseases. Fibrotic diseases can affect various organs and tissues in the body. There is growing evidence that MMT is crucial for the development of fibrotic diseases, including renal, pulmonary, cardiac, subretinal, and other fibrotic conditions (**Table 1**) (7, 50–52). Understanding the role of MMT in these fibrotic diseases is essential for developing effective therapeutic strategies to target this process. MMT modulation is expected to prevent and treat fibrosis by controlling the activation and transformation of macrophages and myofibroblasts in affected tissues.

**Table 1:** Mechanistic studies of Macrophage-Myofibroblast Transformation in various fibrotic diseases.

Disease	Study type	Subjects	Mutagen	Curing	Adverse effects	Ref.
Renal fibrosis	*In vivo* *+* *in vitro*	C57BL/6 mice, BMDMs	UUO surgery, TGF-β1	—	Inhibition of Src effectively blocked MMT in UUO kidneys and macrophages in response to TGF-β1 stimulation, and Src is a direct Smad3 target gene and acts as a key regulator of TGF-β1-mediated MMT.	(54)
		C57BL/6 mice, BMDMs	UUO surgery, TGF-β1	—	Transformation of bone marrow monocyte/macrophage populations derived from the bone marrow into fibroblasts leads to progressive renal tissue fibrosis, an MMT process regulated by TGF-β/Smad3 signalling.	(31)
		C57BL/6 mice, RAW264.7, NRK-52E	UUO surgery, cisplatin or TGF -β1	—	SGK3/TOPK axis activation promotes CD206+ M2 macrophage polarization, causing renal fibrosis by mediating MMT generation.	(59)
		SD rats, RAW264.7 cells	anaerobic treatment	—	Under hypoxic conditions, aldosterone binding to MR stimulates MMT, leading to renal fibrosis. In addition, activation of MR may contribute to MMT, leading to a predominant M1 phenotype under hypoxic conditions.	(43)
		C57BL/6 mice, BMDMs	Intraperitoneal injection of FA, TGF-β1 or IL-4	—	The STING/TBK1 signalling pathway has an important role in bone marrow-derived fibroblast activation, macrophage-to-myofibroblast transformation, and renal fibrosis progression.	(65)
		C57BL/6 mice, BMDMs	UUO surgery, Collection of neutrophils	—	caspase-11/GSDMD-dependent NETs promote renal fibrosis by promoting inflammation and MMT	(66)
Renal fibrosis	*In vivo* *+* *in vitro*	C57BL/6 mice, macrophages	Knockout, folate-induced AKI-CKD transformation model	—	Gsdmd deficiency inhibits NET formation and subsequently prevents NET-induced MMT from occurring.	(67)
		C57BL/6J mice, BMDMs	UUO or IRI surgery, TGF-β1	—	Pou4f1-dependent fibrotic gene network promotes TGF β 1/Smad3-driven MMT in BMDMs at the transcriptional level.	(68)
		C57BL/6 mice, RAW264.7 cells, BMDMs	UUO Surgery, TGF-β1, LY2109761	—	ALD/MR/TGF - β1 signalling pathway induces MMT and is involved in renal fibrosis.	(64)
		C57BL/6J mice, BMDMs	Knockout, IL-4	—	IL-4 stimulates the Jmjd3/IRF4 axis, leading to myeloid fibroblast activation and M2MMT, and renal fibrosis	(70)
		Patients, C57BL/6J mice, RAW264.7 cell, BMDMs	Glyoxalate-induced Calcium oxalate stones, M-CSF	—	STAT6 transcriptionally represses PPAR α and FAO via a cis-inducible element located in the promoter region of the gene, thereby promoting MMT and renal fibrosis.	(71)
		CKD patients, C57BL/6 mice, BMDMs	UUO surgery, M-CSF	—	P2Y12 is highly expressed by macrophages in fibrotic kidneys and promotes MMT-mediated renal fibrosis through TGF - β/Smad3 signalling.	(72)
		C57BL/6 mice, BMDMs, HEK293 cells	UUO Surgery, Lipocalin Compound C or AICAR	—	Inhibition of lipocalin/AMPK signalling may represent a novel therapeutic approach for chronic fibrotic kidney disease.	(24)
		C57BL/6 mice, RAW 264.7 cell	UUO surgery, TGF-β1	—	tFNAs inactivate signal transducers and activators of transcription (Stat) and the TGF-β 1/Smad pathway to regulate M2 polarization and MMT processes.	(40)
		DN rats, RAW 264.7 cells	STZ induced diabetes	VD	VD inhibits the transition of macrophages to the M1 phenotype via the STAT-1/TREM-1 pathway	(79)
Renal fibrosis	*In vivo* *+* *In vitro*	C57BL/6J mice, BMDMs	UUO surgery, M-CSF, TGF-β 1	PTB	PTB mediates MMT to attenuate renal interstitial fibrosis by regulating the transcriptional activity of CXCL10.	(46)
		BALB/c mice, NIH3T3 and RAW 264.7 cells, BMDMs	UUO Surgery, Targeted labelling	NT-NE(C+D)	Targeted nanotechnology was used to codeliver ERS inhibitors and conventional glucocorticoids to precisely modulate the ATF6/TGF-β/Smad3 signalling axis in macrophages. This approach calibrated the levels of TGF-β stimulation on macrophages, promoted their polarization towards the M2c phenotype, and inhibited excessive MMT polarization.	(80)
		Diabetic SD rats, macrophages, PBMCs	Streptozotocin, Adenosine/TGF-β	MRS1754	Pharmacological blockade of A2BAR attenuated some clinical signs of renal insufficiency and glomerulosclerosis in DN rats and reduced intraglomerular macrophage infiltration and MMT.	(81)
		C57BL/6J mice, BMDMs	Gene knockout, UUO surgery, TGF-β1	MBL	MBL inhibits the MMT process by suppressing MMP-9 production and activation of Akt signalling.	(82)
		Renal samples, C57BL6/J mice, BMDMs	UUO surgery, M-CSF and TGF-β 1	FABP4	The causal relationship between FABP4 activation and MMT and the role of FABP4-mediated MMT in the pathogenesis of tissue fibrosis were identified.	(83)
		C57BL/6 mice, RAW264.7 cells, BMDMs	Single nephrectomy, TGF-β1, M-CSF	methadone	Aldosterone promotes renal fibrosis by activating MR and upregulating TGF-β1 expression, mainly inducing M1MMT.	(64)
		C57BL/6 mice, BMDMs	UUO surgery, M-CSF	ICG - 001	Inhibition of β - catenin/TCF by ICG - 001 combined with TGF - β 1 treatment increased β-catenin/Foxo1 and reduced MMT and inflammatory cytokine production in bone marrow-derived macrophages, thereby attenuating renal fibrosis in the UUO model.	(84)
Renal fibrosis	*In vivo*	SD rats, BMDMs	UUO surgery, M-CSF	Epl	Eplerenone attenuates contralateral renal fibrosis in UUO rats by blocking MMT	(45)
		SD rats	UUO surgery	HJHR	HJHR attenuated fibrosis in the contralateral kidney of UUO rats by preventing MMT through the aldosterone/mineralocorticoid receptor/serum/glucocorticoid regulated kinase 1 pathway.	(78)
		Human kidney transplant biopsies, C57BL/6 mice	thalassaemia	—	Myofibroblasts are primarily involved in the transformation of macrophages with a predominantly M2 phenotype and TGF-β/Smad3 signalling is a key regulatory mechanism that promotes MMT and interstitial fibrosis.	(30)
		C57BL/6 mice	UUO surgery	—	Coexpression of cellular antigens identified the MMT process and determined the bone marrow-derived monocyte/macrophage origin of the majority of the myofibroblast population present during renal fibrosis.	(32)
		Patients, mouse model	UUO surgery	—	MMT cells have a predominant M2 phenotype in human and mouse renal fibrosis.	(7)
		C57BL/6 mice	UUO surgery	—	Inhibition of SETD7 inhibits the aggregation of M2MMT and bone marrow-derived myofibroblasts, attenuates the inflammatory response, and inhibits the development of renal fibrosis.	(73)
		C57BL/6 mice	Knockout, UUO surgery	—	The NKT cell/IL-4 signalling pathway stimulates the activation of bone marrow-derived fibroblasts and the conversion of M2-type macrophages to myofibroblasts.	(62)
		C57BL/6 mice	UUO surgery	—	BRP-39 is an important activator of macrophage-myofibroblast crosstalk and pro-fibrotic signalling.	(74)
Pulmonary fibrosis	*In vivo* *+* *In vitro*	C57BL/6 mice, A549, BMDMs	Subcutaneous injection of LLC, TGF-β1, IL - 4	—	Macrophage-specific silencing of Smad3 effectively blocks MMT, thereby inhibiting CAF-mediated cancer progression.	(86)
		C57BL/6 mice, MH-S cells	SiO_2_	quercetin	SiO_2_ activates macrophage polarity and MMT via the TGF-β-Smad2/3 signalling pathway. quercetin also attenuates MMT and TGF-β Smad2/3 signalling pathways *in vivo* and *in vitro*.	(85)
	*In vivo*	C57BL/6 mice	UUO surgery	eplerenone	Cells that develop MMT make up a significant part of the myofibroblast population and are associated with pulmonary fibrosis, with a predominantly M2 phenotype in the lungs and attenuated after eplerenone treatment.	(52)
	*In vitro*	*Macaca mulatta* lung tissue section samples, GranSim framework for virtual granuloma modelling	—	—	For MMT to contribute to granuloma-associated fibrosis, it must be driven by sequential stimulation of M1 macrophages with anti-inflammatory signalling and inhibited by excessive inflammatory/inflammatory signalling and *Mycobacterium tuberculosis* load.	(42)
		MH-S cells	rapamycin	—	Macrophages exposed to TGF-β have the potential to transdifferentiate into myoblasts, a process in which CCR8 is involved and which occurs mainly through autophagy.	(87)
Cardiac fibrosis	*In vivo* *+* *In vitro*	Wistar rats, RAW264.7 cells	UUO surgery, TGF-β 1	eplerenone	Eplerenone inhibits UUO-induced MMT in rats with type 4 cardiorenal syndrome via the MR/CTGF pathway.	(93)
		C57BL/6 mice, Primary macrophages	gene knockout, high-fat diet + Ang II, Ang II	—	The ALKBH5/IL-11/IL11RA1/MMT axis alters cardiac macrophages and leads to fibrosis and dysfunction in hypertensive hearts of mice, thus identifying a potential target for cardiac fibrosis treatment in patients.	(95)
Cardiac fibrosis	*In vivo* *+* *In vitro*	C57BL6/J mice, monocytes	Myocardial infarction surgery, PMA (induced macrophage)	—	Macrophage depletion markedly decreased the count of Mac3+ Col1A1+ cells in the heart following myocardial infarction, and therapeutic control of MMT might be anticipated to positively influence the fibrotic reaction postmyocardial infarction and other cardiovascular pathological conditions	(96)
		C57BL/6 mice, Primary PBMCs, BMDMs	Myocardial infarction surgery, LPS, IL-4, TGF-β	—	When the tissue environment changes from proinflammatory to reparative, S100a9 activates TGF-β/p-smad3 signalling to induce MMT and promote myocardial fibrosis.	(97)
Retinal fibrosis	*In vivo* *+* *In vitro*	Human eye samples, C57BL/6J mice, BMDMs	Laser, TGF-β 1	—	MMP12 plays a key role in the development of subretinal fibrosis, in part by promoting MMT.	(101)
		Vitreous fluid samples from PDR patients, MIO-M1, HRMECs	(w/v) Gelatin	—	The process of MMT may contribute to the formation of myofibroblasts in the anterior retinal membrane, a transition that involves macrophages with a predominantly M2 phenotype.	(102)
		Human eye samples, C57BL/6J mice, BMDMs	Laser, TGF-β1	—	MMT plays a role in macular fibrosis secondary to nAMD.TGF-β and C3a induce MMT, whereas C5a does not.	(51)
Liver fibrosis	*in vivo*	Human liver specimens, C57BL/6 mice	Intraperitoneal injection of carbon tetrachloride	—	MMT cells have a predominantly M2 phenotype in human and experimental chronic liver injury.	(50)
Pancreatic fibrosis	*in vitro*	Primary human CD14^+^ monocytes	H_2_O_2_ or radiation	—	Oxidative stress Oxidative stress induces MMT through activation of the p38-MAPK pathway, which induces reactive substrates.	(104)
Skeletal muscle fibrosis	*In vivo* *+* *in vitro*	C57/6J mice, BMDMs	analgesia	—	C3a stimulation directly induces MMT in BMDMs.	(105)
Incision	*In vivo* *+* *in vitro*	C57BL/6 mice, Human samples, HaCaT cell	excision	—	EVs from wound fluids from patients with healing chronic wounds were enriched in miR-21 and more efficiently induced the transformation of myeloid cells to fibroblast-like cells compared to fluids from unhealed patients.	(41)

### 4.1 Renal fibrosis

Renal fibrosis is a crucial pathological biomarker of chronic renal disease (CKD). It is characterized by the buildup of excessive ECM, which replaces healthy tissue and eventually results in end-stage renal disease (53). The medullary lineage of MMT cells plays a substantial role in the myofibroblast population, and the MMT process is a broad mechanism of renal interstitial fibrosis (6). A study revealed that BMDMs can transform into collagen-producing myofibroblasts in chimeric mice with renal fibrosis, suggesting that myofibroblasts may have nonrenal origins (29). This study identified a gene network centred on Src (sarcoma gene) as a potential mechanism of MMT by RNA sequencing, ultimately revealing Src as a key regulator of MMT. Src was found to participate in the MMT process as a target gene of smad3 (3, 54). Inhibition of Src *in vivo* and *in vitro* effectively reduced the MMT process (54, 55). Research indicates that MMT promotes renal interstitial fibrosis in mice and humans (30). TGF-β1 plays a crucial role in suppressing inflammation and facilitating tissue healing, but it also contributes to the development of chronic fibrotic conditions (56, 57). MMT specifically occurs in fibrotic renal tissue and is controlled by TGF-β/Smad3 signalling (31). Aldosterone acts on renal vasculature and cells, induces SGK-1 expression, activates NF-κB, and plays an important role in MMT-induced contralateral renal fibrosis (58). TGF-β1 promotes the upregulation of the macrophage SGK3/TOPK signalling pathway following renal injury to enhance M2 macrophage conversion and MMT. This process accelerates the progression of AKI (acute renal injury) to CKD (chronic renal disease) (59). In the early stage of AKI, M1 macrophages predominate in renal tissue and play a proinflammatory role. Subsequently, M2 macrophages replace M1 macrophages and primarily exert anti-inflammatory effects to promote renal repair (37, 60). However, the persistence of M2 macrophages leads to the development of MMT, which promotes renal fibrosis (3, 61). The NKT cell/IL-4 signalling pathway stimulates activation of bone marrow-derived fibroblasts and conversion of M2 macrophages to myofibroblasts in renal fibrosis (62). *In vivo* and *in vitro* results have shown that the ALD/MR/TGF-β1 signalling pathway induces MMT and is involved in renal fibrosis (63, 64). The interferon gene-stimulating factor/TANK-binding kinase 1 (STING/TBK1) axis is a major regulator of the innate immune response and plays a key role in bone marrow-derived fibroblast activation, MMT, and renal fibrosis progression (65). The study found that Caspase-11/GSDMD-mediated neutrophil extracellular traps (NETs) stimulate macrophages to produce inflammatory cytokines, promoting inflammation through NF-κB nuclear translocation. NETs can also drive MMT and, consequently, renal fibrosis via the TGF-β1/Smad3 signalling pathway (66). GSDMD deficiency inhibited NETs formation and impeded NETs-induced MMT (67). A brain-specific transcription factor, Pou4f1, was identified as a key regulator of TGF-β1/Smad3 signalling in MMT. Knocking down Pou4f1 in BMDMs prevents infiltrating renal macrophages from undergoing MMT, resulting in a notable decrease in renal fibrosis (68). IRF-4 deficiency impedes the inflammatory response, activation of bone marrow-derived fibroblasts, and MMT in nephropathy (69). There are also relevant studies the Jmjd3/IRF4 signalling pathway is crucial for activating bone marrow fibroblasts, promoting MMT, depositing extracellular matrix proteins, and advancing renal fibrosis (70). STAT6 contributes to renal fibrosis by interacting with the PPARα promoter, suppressing FAO, and supporting MMT (71). Macrophages in the fibrotic kidneys of CKD patients and unilateral ureteral obstruction (UUO) model mice exhibit high P2Y12 expression. TGF-β1 induces P2Y12 via a Smad3-dependent mechanism, thereby promoting MMT-mediated renal fibrosis (72). Inhibition of SETD7 suppresses MMT and myeloid-derived myofibroblast aggregation, reduces the inflammatory response, and inhibits the progression of renal fibrosis (73). BRP-39 plays a crucial role in macrophage-myofibroblast crosstalk and necrotic signalling for inadequate renal repair (74). Studies have demonstrated the involvement of both the adiponectin/APK and JAK3/STAT6 signalling pathways in the development of experimental renal fibrosis and collagen production by cultured mouse cells undergoing MMT (24, 75). Myofibroblast-derived exosomes enhance MMT and renal fibrosis while inhibiting exosome production, leading to reduced collagen deposition, extracellular matrix protein accumulation, and MMT in FA nephropathy (76). Conversely, treatment with human umbilical cord MSC-derived exosomes significantly diminishes kidney injury and fibrosis in UUO mice, downregulating fibrosis markers and inhibiting MMT (77).

**Figure 2:**
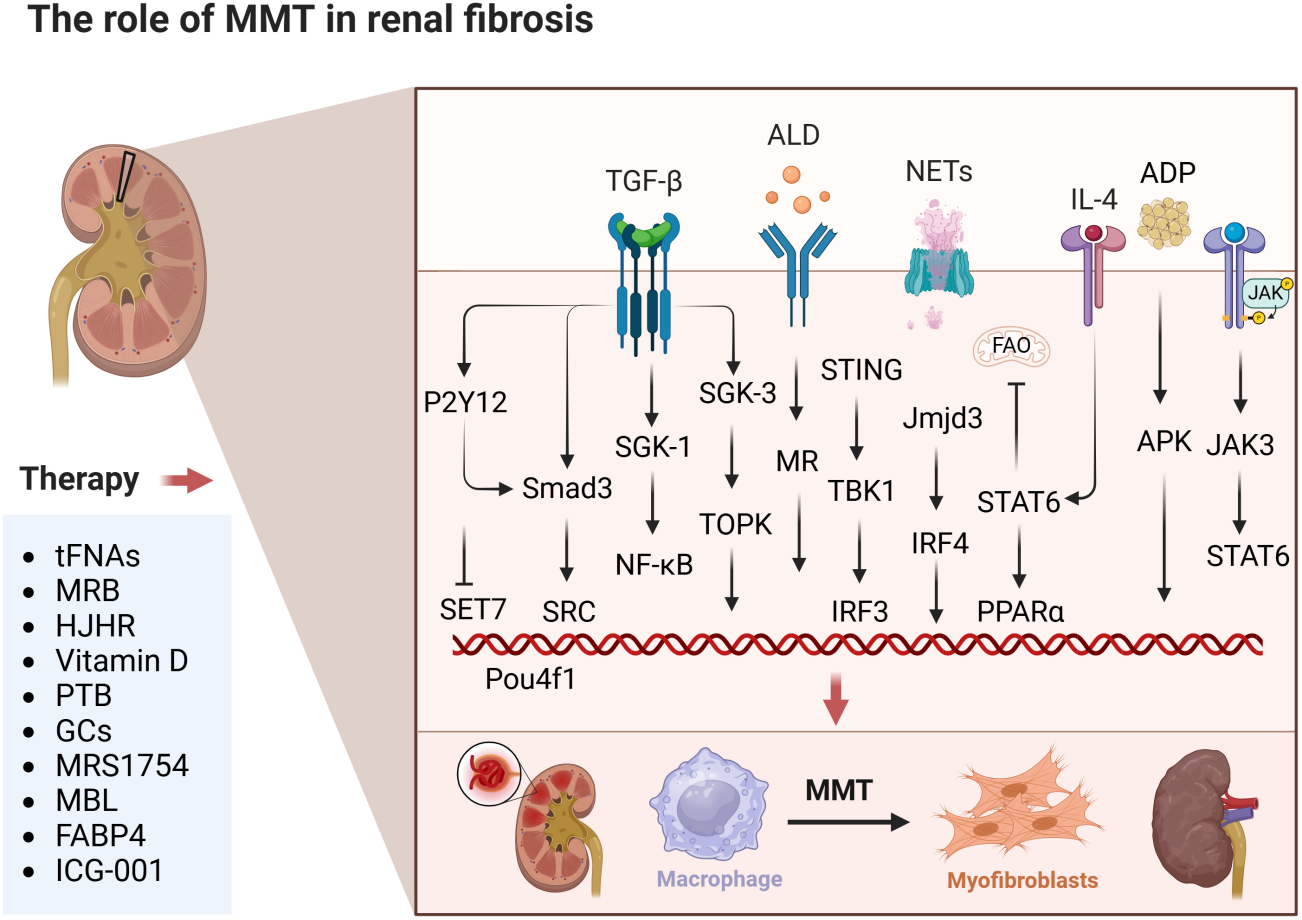
The role of MMT in renal fibrosis. The figure focuses on the specific mechanisms of occurrence and effects of MMT in kidneys. This includes drug treatment strategies in experimental articles. The above therapeutic strategies are summarised diagrams, with each drug having a corresponding target for intervention. The details are clearly indicated in the list below. Created with BioRender.com.

Currently, pharmacological intervention in the MMT process is available for renal fibrosis studies. TFNAs, which are tetrahedral framework DNA nanostructures, can improve bladder fibrosis and dysfunction caused by bladder outlet obstruction (BOO) by preventing M2 macrophage polarization and mesenchymal-to-mesenchymal transition processes. The inhibitory effects of tFNAs on M2 polarization and MMT processes are due to the deactivation of STAT and TGF-β1/Smad signalling mechanisms (40). Eplerenone attenuates contralateral renal fibrosis in UUO rats by blocking MMT (45). Aldosterone (ALD) stimulated macrophages to express higher levels of α-SMA, indicating enhanced MMT. These effects were significantly blocked by HJHR (the Huoxue Jiedu Huayu recipe). HJHR inhibited MMT by inhibiting the ALD/MR/SGK-1 signalling pathway, attenuating interstitial fibrosis in obstructive nephropathy (78). Vitamin D inhibits the shift of macrophages to the M1 phenotype via the STAT-1/TREM-1 pathway. It also enhances the M2 macrophage phenotype and prevents cell damage (79). Pterostilbene (PTB) attenuated renal interstitial fibrosis in UUO mice by mediating MMT (46). This research employed specific nanotechnology to simultaneously administer endoplasmic reticulum stress (ERS) inhibitors (ceapin 7, Cea, or C) and traditional glucocorticoids (dexamethasone, Dex, or D) to regulate the ATF6/TGF-β/Smad3 signalling pathway in macrophages with precision. This method adjusted the TGF-β activation on macrophages, encouraged their transformation into the M2 phenotype, and prevented excessive MMT polarization (80). MRS1754 blockade of A_2B_ AR attenuated renal insufficiency and reduced intraglomerular macrophage infiltration and MMT in rats (81). MBL (mannose-binding lectin) inhibited the MMT process by suppressing MMP-9 production and activating Akt signalling (82). Fatty acid binding protein 4 (FABP4) inhibited the MMT process in TGF-β1-stimulated BMDMs from IgAN patients and UUO model mice. In this process, serum amyloid A1 (Saa1) is a gene directly regulated by FABP4 in the MMT process of renal fibrosis (83). Esaxerenone inhibits MMT through the mineralocorticoid receptor/TGF-β1 pathway in mice induced with aldosterone (64). ICG-001 inhibited the β-catenin/TCF interaction, reducing MMT in bone marrow-derived macrophages during renal fibrosis and enhancing the anti-inflammatory effect of TGF-β on bone marrow-derived macrophages, leading to reduced renal fibrosis in the UUO model (84) (**Figure 2**).

Most of the studies on the role of MMT in the mechanism of renal fibrosis have focused on the upstream and downstream signals activated by the TGF-β/Smad signalling pathway. For example, its upstream signalling factors Src, SGK-1/NF-κB, caspase-11/GSDMD, Pou4f1, ALD/MR/SGK-1, β-catenin/TCF, P2Y12, Stat, ATF6, Saa1, and its downstream signalling factors are mainly SGK3/TOPK. This specific research is still in progress. The remaining signalling pathways mediating renal fibrosis are STING/TBK1, IL-4Ra/STAT6, Jmjd3/IRF4, STAT6/PPARα, SETD7, NKT/IL-4, BRP-39, STAT-1/TREM-1, MMP-9/Akt, adiponectin/APK, and JAK3/STAT6. present. The main pharmacological interventions for attenuating renal fibrosis through MMT are tFNAs, HJHR, vitamin D, PTB, ERS inhibitors, MRS1754, MBL, and FABP4. However, the exact mechanisms and factors that regulate the MMT in renal fibrosis are not yet fully understood, and this topic remains an active area of research. Although it is clear that macrophages play a key role in renal fibrosis, the exact role and intrinsic mechanisms of MMT are still being explored.

### 4.2 Pulmonary fibrosis

Pulmonary fibrosis is characterized by excessive extracellular matrix accumulation, which leads to a decrease in lung function. Recent evidence indicates that MMT plays a key role by increasing myofibroblast numbers and promoting fibrotic lung tissue remodelling. Single-cell RNA sequencing and fluorescence *in situ* hybridization studies have shown the coexpression of monocyte/macrophage and myofibroblast markers in patients with fibrotic lung disease. Approximately 35% of myofibroblasts in fibrotic lung tissues express the M2 macrophage marker CD206, suggesting potential M2 transformation before myofibroblasts change (12). UUO-induced renal injury activates ALD and mineralocorticoid receptors, triggering MMT in the lungs and resulting in lung injury and fibrosis (52). TGF-β-Smad2/3 signalling is crucial for macrophage transformation in lung fibrosis (85). Macrophage-specific Smad3 silencing effectively hinders MMT and may prevent disease progression (86). Chemokine receptor 8 (CCR8) promotes lung fibroblast formation via MMT by enhancing autophagy, significantly increasing TGF-β-induced cell migration and the expression of waveform protein and α-SMA in macrophages (87). MMT promotes the development of peripheral fibrosis in TB granulomas by influencing fibroblasts rather than causing fibrosis directly. For MMT to contribute to granuloma-related fibrosis, it must be activated by sequential stimulation of M1 macrophages with anti-inflammatory signals and inhibited by hyperinflammatory/inflammatory signals (42). This study suggested that to promote the MMT process, resting macrophages are forced to receive STAT1 and STAT3 signals through appropriate pathways, leading them to immediately switch phenotypes to become myofibroblasts (42). This indicates that STAT1 and STAT3 could be responsible for promoting MMT in living organisms, but further experiments are needed for full exploration and validation. The functional phenotype of macrophages at the injury site extends beyond switching from the proinflammatory M1 state to the anti-inflammatory M2 state (88).

Currently, research on the treatment of fibrosis involves the use of several interventional drugs to inhibit the process of MMT. Early treatment with quercetin attenuates the macrophage transformation process and can reduce macrophage polarization during pulmonary fibrosis (85). Studies have also confirmed that the MR blocker eplerenone prevents UUO-induced pulmonary fibrosis (52). Eplerenone reduces the accumulation of MMT cells in the lungs, macrophages are one of the target cells of salt corticosteroids, and aldosterone may directly affect macrophage phenotypic transformation (89, 90). There is increasing evidence that spironolactone attenuates acute lung injury and fibrosis by inhibiting MR-mediated phenotypic conversion of circulating monocytes and alveolar macrophages (91).

**Figure 3:**
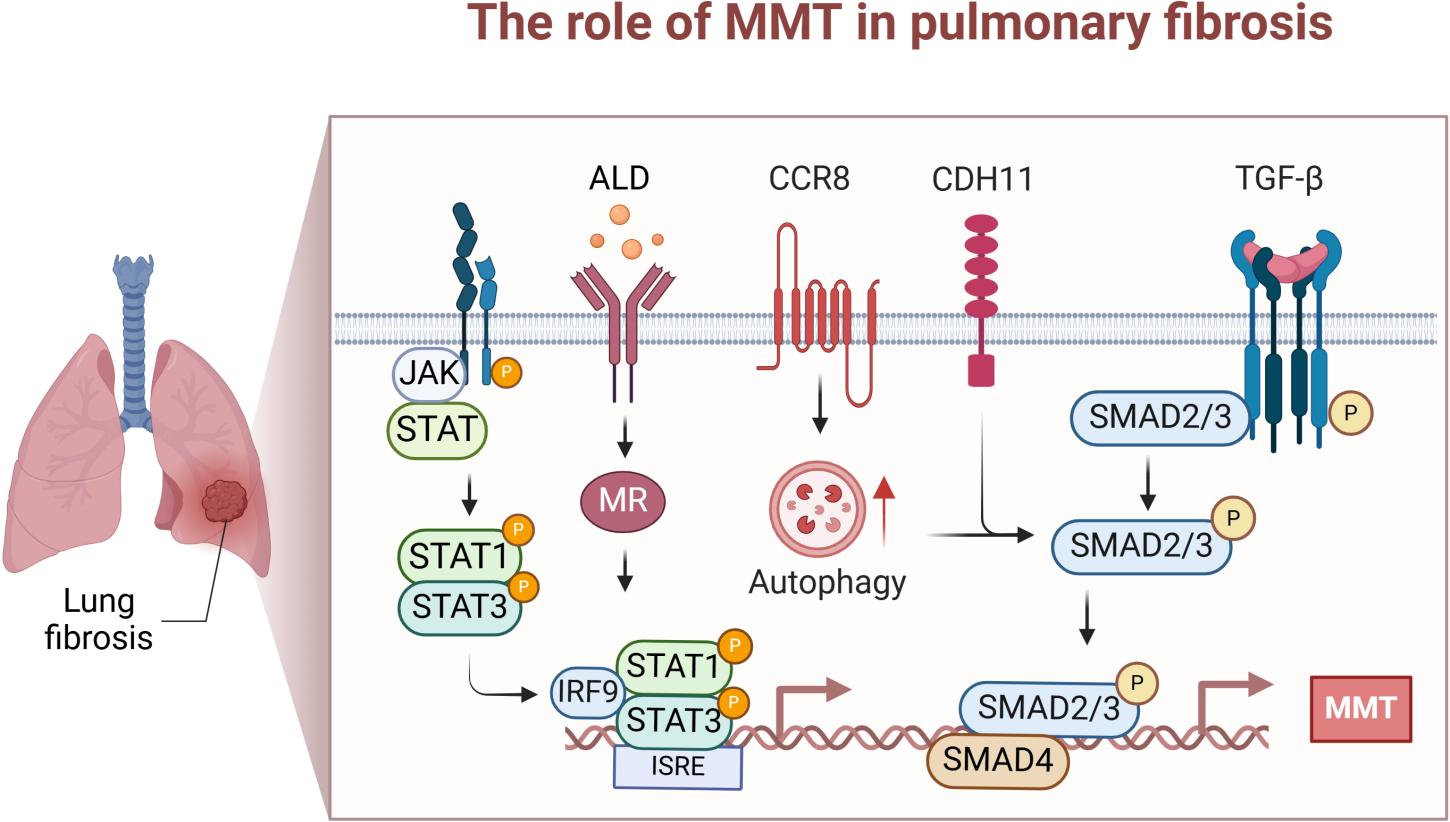
The role of MMT in lung fibrosis. This diagram focuses on the specific mechanisms and effects of MMT occurrence in the lung. These mainly include STAT1 and STAT3 driving MMT; ALD/MR in the kidney inducing intrapulmonary MMT; and Chemokine receptor 8 (CCR8) promoting TGF-β/Smad-induced MMT through enhanced autophagy. Created with BioRender.com.

To date, studies on the role of MMT in pulmonary fibrosis are limited. Taken together, reducing the process of MMT or interfering with critical mediators (e.g., TGF-β-Smad2/3, CDH11, CCR8, autophagy, STAT1, and STAT3 signalling) may help to prevent or reverse fibrotic remodelling. The main therapeutic agents studied for lung fibrosis are quercetin, the MR blocker eplerenone, and spironolactone, all of which may attenuate lung fibrosis by reducing the effect of MMT. Although the role of MMT in pulmonary fibrosis is increasingly recognized, there are still many gaps in our understanding. For example, the specific triggers and signalling pathways that drive MMT require further investigation. In addition, the heterogeneity of macrophage populations and their contributions to MMT at different fibrosis stages remain to be elucidated. The MMT process appears to be critical for myofibroblast aggregation and fibre remodelling in the lung. Further studies are needed to deepen our understanding of the complex mechanisms of MMT and identify potential therapeutic targets for treating pulmonary fibrosis (**Figure 3**).

### 4.3 Cardiac fibrosis

**Figure 4:**
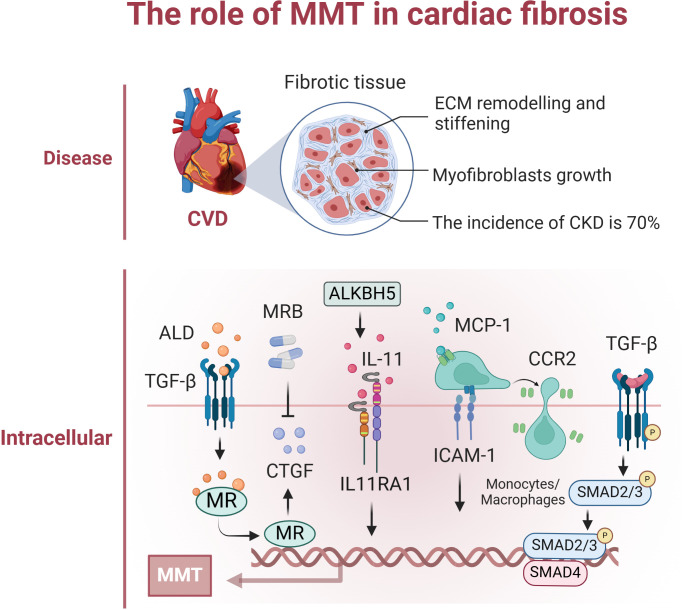
The role of MMT in cardiac fibrosis. The figure focuses on the specific mechanisms of occurrence and effects of MMT in cardiac. This mainly included renal-secreted ALD promoting cardiac MMT; UUO induced high expression of MCP-1, CCR2, and ICAM-1, which regulated macrophage infiltration in the heart and promoted MMT; and the ALKBH5/IL-11/IL11RA1/MMT axis altered cardiac macrophages promoting MMT. Created with BioRender.com.

The prevalence of cardiovascular disease (CVD) in patients with chronic renal disease (CKD) is approximately 70%, which is almost double the prevalence of CVD in the non-CKD population. Furthermore, approximately 50% of all deaths associated with renal illness are attributed to cardiovascular disease (92). UUO causes damage to the heart and leads to the formation of scar tissue. Aldosterone stimulation of MR enhances macrophage production of connective tissue growth factor (CTGF), leading to MMT and consequent cardiac fibrosis. Eplerenone decreases CTGF overexpression, indicating that inhibiting the MR/CTGF pathway with eplerenone could be a promising approach for managing myocardial fibrosis (93). CTGF functions as a mediator following TGF-β1 signalling, and inhibiting CTGF reduces the effects of TGF-β1 and aldosterone on MMT. UUO significantly increased the expression of MCP-1, CCR2, and ICAM-1, which are essential chemokines that control the invasion and adherence of monocytes/macrophages in the rat heart (94). Activation of the ALKBH5/IL-11/IL11RA1 pathway triggers MMT onset, changes cardiac macrophages, and induces fibrosis and dysfunction in hypertensive mouse hearts. This discovery highlights a promising target for treating cardiac fibrosis in patients. Targeting the ALKBH5-IL-11-IL11RA1 pathway in cardiac macrophages using LNPsiRNA technology could be a valuable therapeutic approach for preventing cardiac fibrosis in heart disease patients (95). Macrophage depletion markedly decreased the number of Mac3^+^ Col1A1^+^ cells in the heart following myocardial infarction, and therapeutic control of MMT might positively influence the fibrotic reaction postmyocardial infarction and other cardiovascular pathological conditions (96). Research has demonstrated that single-cell RNA sequencing (scRNA-seq) may identify a specific type of macrophage produced from monocytes that heavily invade injured heart tissue during acute MIR, and these macrophages exhibit high levels of S100a9 expression. S100a9hi macrophages can stimulate fibroblasts to differentiate into myofibroblasts via the TGF-β/p-Smad3 signalling pathway, promoting myocardial fibrosis through MMT (97). Fewer studies are related to the MMT process in cardiac fibrosis, and progress is still needed. Preventing the progression of cardiac fibrosis may help to attenuate death associated with renal disease, as well as prevent cardiovascular complications and related deaths (**Figure 4**).

### 4.4 Retinal fibrosis

Proliferative vitreoretinal disorders are characterized by inflammation and fibrosis. Subretinal fibrosis is a vascularized lesion characterized by a high presence of immune cells and myofibroblasts and excessive extracellular matrix protein deposition, typical of “thermal fibrosis,” unlike fibrotic scarring, which aids in healing skin wounds (98, 99). “Thermofibrosis” involves dynamic interactions between myofibroblasts and macrophages (immune cells), causing ongoing inflammatory damage to healthy cells such as retinal pigment epithelial (RPE) cells and photoreceptors, ultimately resulting in irreversible vision loss (100). This study utilized RNA sequencing to identify MMP12, often referred to as macrophage elastase, as one of the most prominently elevated genes in subretinal fibrosis. Subsequent *in vitro* and *in vivo* research demonstrated the involvement of MMP12 in TGFβ-induced MMT, and inhibiting MMP12 notably reduced subretinal fibrosis (101). Monocytes that are translocated to myofibroblasts during MMT need to be recruited into the ocular microenvironment, where they form myofibroblasts in the retinal membrane, a transition involving predominantly M2 macrophages (102). In addition, fibroblast-activated protein-interacting network α (FAP-α), a vitreous biomarker, is derived from the conversion of M2 macrophages and Müllerian glial cells to myofibroblasts. Müllerian glial cells are the predominant cell type involved in inflammatory responses in the retina (103). TGF-β could play a crucial role in promoting epithelial–mesenchymal transition in retinal fibrosis. Research indicates that the MMT plays a role in macular fibrosis associated with neovascular age-related macular degeneration (nAMD). Approximately 5-10% of macrophages treated with C3a expressed α-SMA, fibronectin, and collagen-1. This process, known as C3a-mediated MMT, can be inhibited by the C3aR antagonist C3aRA (51).

### 4.5 Other types of fibrosis

Liver fibrosis is a pathophysiological reaction to chronic liver damage caused by factors such as viral infection, medication toxicity, and alcoholic or nonalcoholic fatty liver disease. In clinical liver fibrosis tissue samples and various animal models of chronic liver injury, macrophages were observed to undergo MMT, indicating the significant involvement of MMT in chronic liver injury and liver fibrosis (50).

Transcriptome analysis revealed that monocytes cultured from patients with pancreatic ductal adenocarcinoma (PDAC) exhibit fibrosis and that monocytes cultured in the presence of hydrogen peroxide (H_2_O_2_) exhibit upregulation of the p53 pathway, suggesting that MMT is induced through the production of stable p53 by reactive oxygen species (104). *In vitro* studies have shown that oxidative stress activates the p38-MAPK pathway and promotes MMT (104).

**Figure 5:**
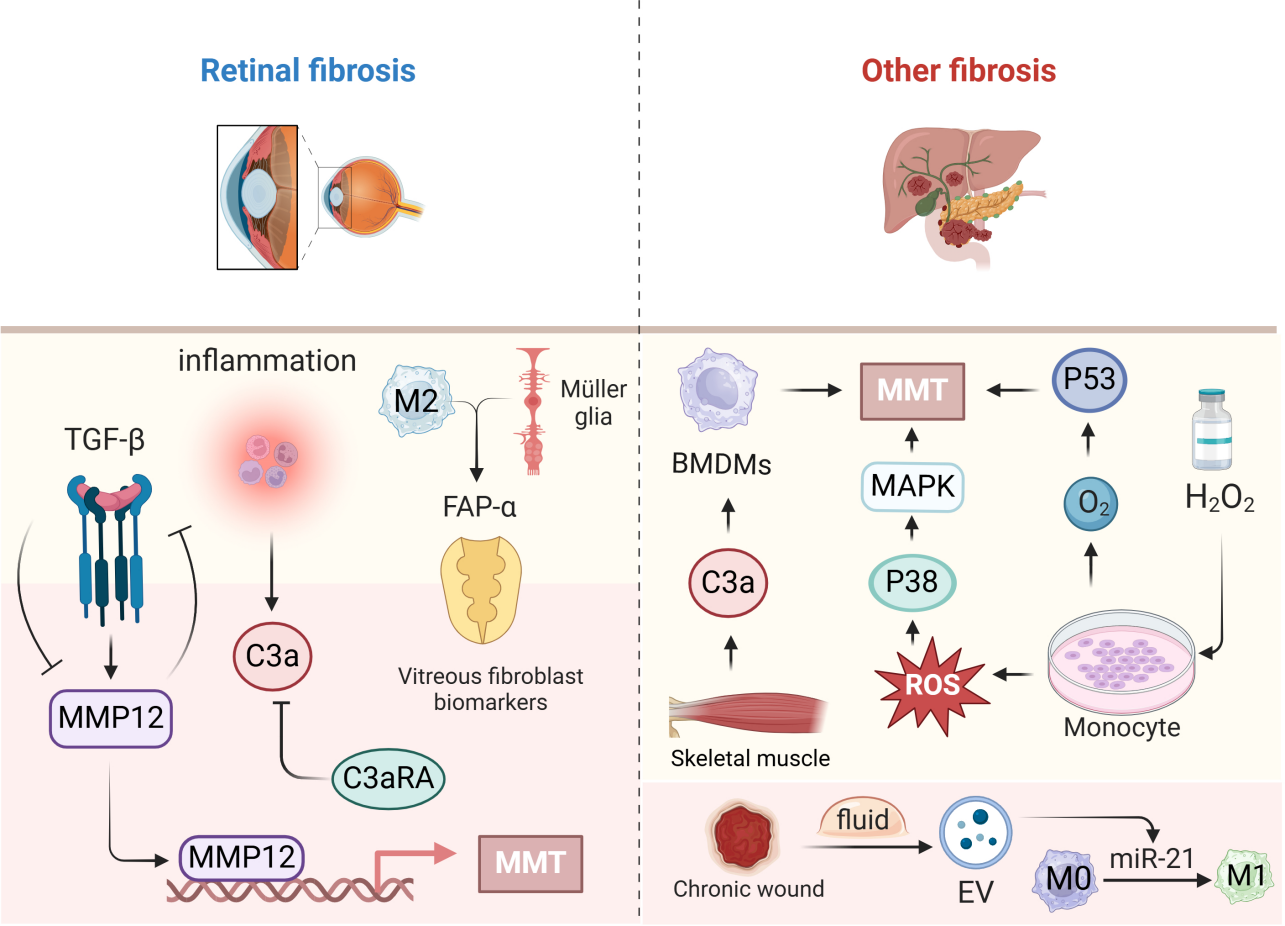
The role of MMT in other fibrosis. This figure focuses on fibrosis due to MMT in the retina on the left side, and on the right side, the specific mechanisms and roles of MMT occurring in other organs such as the liver, pancreas, skeletal muscle, and incisions. Created with BioRender.com.

Acute skeletal muscle injury is commonly associated with sports and trauma. However, skeletal muscle fibrosis is inevitable after injury. C3ar1, C1qa, C1qb, and C1qc were still notably elevated in MMT cells, indicating a dual function for complement signalling pathways, notably the C3a-C3ar1 pathway, in skeletal muscle repair. After acute skeletal muscle injury, the complement system and associated pathways are activated by MMT, and C3a alone can induce MMT in BMDMs and increase collagen synthesis (105). When peripheral BMDMs are polarized into M1 macrophages, the EVs in wound fluid from patients with healing chronic wounds are enriched in miR-21 and more efficiently induce cellular transformation. At that point, MMT has been shown to occur *in vitro* (41). The study of MMT in other organs is still pending (**Figure 5**).

## 5 Conclusions and outlook

The investigation of macrophage-to-myofibroblast transformation is a prominent area that has recently garnered significant interest. This process involves both phenotypic alterations and functional shifts in cells and has crucial biological implications and potential clinical uses. Ongoing research indicates that macrophages can transition phenotypically into myofibroblast-like cells under specific circumstances. This cellular metamorphosis not only aids in comprehending the molecular mechanisms governing cell fate but also offers novel insights and approaches for treating certain diseases.

TGF-β1 is a key player in fibre formation and is crucial for promoting the formation of fibres such as collagen. Most studies on MMT focus on the TGF-β/Smad signaling pathway, with only a few exploring other pathways. While the kidney is the primary site of MMT research, MMT in the kidneys can further promote fibrosis in other organs. Additionally, MMT in different organs may occur through various pathways, potentially influenced by the distinct sources of primary macrophages in each organ or the impact of foreign macrophages following injury. TGF-β1 is a multifunctional cytokine that affects various cell types and tissues. But blocking or inhibiting TGF-β1 can have complex and context-dependent effects on fibre formation. There is an urgent need to develop targeted and effective inhibitor therapeutic strategies for fibrosis, as current therapeutic options are limited. Macrophages play dual roles in fibrotic diseases. While they are essential for the initial immune response and tissue repair, their prolonged activation and transformation into myofibroblasts can lead to excessive fibrosis. However, further studies are needed to understand the factors regulating macrophage behaviour and their contribution to fibrotic disease progression. Targeting MMT as a potential treatment for fibrotic diseases is an emerging field, with researchers exploring strategies to regulate MMT through targeted inhibition using drugs, gene therapy, or biologics to halt the transition of macrophages to myofibroblasts. By inhibiting MMT, it is hoped that the progression of fibrosis can be slowed or even stopped.

Future research should investigate whether resident fibroblasts in various organs are the sole contributors to peripheral granulomatous fibrosis, or if other cell types and macrophages from different pathways transform into myofibroblasts. Additionally, the specific macrophage phenotype from which MMT cells originate, as well as the other promoters or upstream triggers and downstream mechanisms of MMT, remain unclear.

Overall, the intensive study of MMT in fibrotic diseases has enhanced our understanding of the underlying mechanisms and potential therapeutic perspectives of MMT. Subsequent studies hold promise for advancing our grasp of fibrosis and aiding in the creation of novel therapeutic approaches. Despite being in its early stages, the realm of macrophage transformation to myofibroblasts shows substantial developmental potential. Its significant involvement in physiological and pathological processes could be further scrutinized in the future, revealing new avenues for cell therapy and regenerative medicine advancement.
